# Laminin and Fibronectin Treatment Leads to Generation of Dendritic Cells with Superior Endocytic Capacity

**DOI:** 10.1371/journal.pone.0010123

**Published:** 2010-04-19

**Authors:** Samuel García-Nieto, Ramneek K. Johal, Kevin M. Shakesheff, Mohamed Emara, Pierre-Joseph Royer, David Y. S. Chau, Farouk Shakib, Amir M. Ghaemmaghami

**Affiliations:** 1 Allergy Research Group, School of Molecular Medical Sciences and Respiratory Biomedical Research Unit, University of Nottingham, Nottingham, United Kingdom; 2 Tissue Engineering Group, School of Pharmacy, University of Nottingham, Nottingham, United Kingdom; Fundação Oswaldo Cruz, Brazil

## Abstract

**Background:**

Sampling the microenvironment at sites of microbial exposure by dendritic cells (DC) and their subsequent interaction with T cells in the paracortical area of lymph nodes are key events for initiating immune responses. Most of our knowledge of such events in human is based on *in vitro* studies performed in the absence of extracellular matrix (ECM) proteins. ECM in basement membranes and interstitial spaces of different tissues, including lymphoid organs, plays an important role in controlling specific cellular functions such as migration, intracellular signalling and differentiation. The aim of this study was, therefore, to investigate the impact of two abundant ECM components, fibronectin and laminin, on the phenotypical and functional properties of DC and how that might influence DC induced T-cell differentiation.

**Methodology/Principal Findings:**

Human monocyte derived DC were treated with laminin and fibronectin for up to 48 hours and their morphology and phenotype was analyzed using scanning electron microscopy, flow cytometry and real time PCR. The endocytic ability of DC was determined using flow cytometry. Furthermore, co-culture of DC and T cells were established and T cell proliferation and cytokine profile was measured using H^3^-thymidine incorporation and ELISA respectively. Finally, we assessed formation of DC-T cell conjugates using different cell trackers and flow cytometry. Our data show that in the presence of ECM, DC maintain a ‘more immature’ phenotype and express higher levels of key endocytic receptors, and as a result become significantly better endocytic cells, but still fully able to mature in response to stimulation as evidenced by their superior ability to induce antigen-specific T cell differentiation.

**Conclusion:**

These studies underline the importance of including ECM components in *in vitro* studies investigating DC biology and DC-T cell interaction. Within the context of antigen specific DC induced T cell proliferation, inclusion of ECM proteins could lead to development of more sensitive assays.

## Introduction

Dendritic cells (DC) are specialized antigen presenting cells which serve as sentinels that capture and carry antigens to local lymph nodes (LN) [1], [2], [3]. In the LN they process and present antigens in association with MHC class II to specific T cells. T helper (Th) cells that have been activated by DC will develop into functionally distinct cell subsets such as Th1, Th2, Treg or Th17 [4]. Polarization towards these effector T cell subsets is critical for defence against invading pathogens, but under pathological conditions could also be associated with the induction of autoimmune (Th1, Th17) or allergic (Th2) diseases. The immunological outcome of antigen presentation by DC to T cells depends on many factors such as DC lineage, the nature of the antigen they come into contact with and the state of DC maturation [3], [5], [6].

Most of our knowledge on the role of human DC in the processing and presentation of antigens to naïve T cells is based on *in vitro* studies, performed in traditional cell culture systems and in the absence of extracellular matrix (ECM) proteins, in which DC are pulsed with pathogen extracts or infected with pathogens and are then co-cultured with naïve T cells [5], [6], [7], [8], [9]. Although these approaches have provided considerable insights into human DC biology, they tend to suffer from the limitations of using conventional cultures, notably the absence of ECM. The presence of ECM and the 3D structure of lymphoid organs are known to play an important role in DC-T cell interaction [10], [11]. For example, the 3D structure of a lymph node ensures targeted positioning of interacting cells, facilitates T cell migration towards DC, supports motility upon cell-cell interaction and provides traction for amoeboid T cell crawling within the DC compartment. Furthermore, DC-T cell interaction takes place in the presence of ECM, the natural medium in which cells proliferate, differentiate and migrate. Cell-ECM interaction is specific and bi-univocal and controls and guides specific cell functions such as migration, proliferation, intracellular signalling and differentiation [10], [11], [12], [13]. In this context, ECM has been shown to prevent passive cell aggregation, and under those conditions T cell crawling is likely to occur at the interface between the DC membrane and ECM components [14].

In an attempt to better simulate these *in vivo* events, some investigators have resorted to studying DC-T cell interaction in a collagen lattice [15], [16], but given that only a very small amount of collagen is actually available within the paracortical region of LN [17], where DC-T cell interaction takes place, the physiological relevance of using collagen in this context is questionable.

Given the abundance of extracellular matrix proteins *in vivo*, potential influence of ECM on DC differentiation from progenitor cells is highly likely. Indeed Staquet and colleagues [18] have shown that fibronectin could aid the differentiation of dendritic cells from CD34^+^ progenitor cells and fragments of the fibronectin protein have been shown to induce the differentiation of monocyte derived dendritic cells [19].

Thus, the aim of this study was to investigate the impact of fibronectin [FN] and laminin [LMN], two major ECM components that are abundant in the LN paracortex [20] as well as interstitial spaces, on the phenotypical and functional properties of DC and how that might influence DC induced T-cell differentiation. Here we show that in the presence of ECM, DC maintain a ‘more immature’ phenotype and express higher levels of key endocytic receptors, and as a result become significantly better endocytic cells, but still fully able to mature in response to stimulation as evidenced by their superior ability to induce antigen-specific T cell differentiation.

## Materials and Methods

### Ethics Statement

For generating monocyte derived dendritic cells we used peripheral blood of healthy volunteers which was obtained with prior written consent and The University of Nottingham Medical School Research Ethics Committee approval.

### Generation of DC

DC were generated from peripheral blood monocytes as described before [21]. Briefly, PBMCs were separated from heparinized peripheral blood of healthy volunteers by standard density gradient centrifugation on Histopaque (Sigma, Irvine, UK). Monocytes were isolated from PBMCs using CD14 microbeads (Miltenyi Biotec, Bergisch Gladbach, Germany) and cultured in RPMI medium (Sigma) supplemented with L-glutamine, antibiotics and 10% fetal calf serum (FCS) containing 50 ng/ml of GM-CSF and 250 U/ml of IL-4 (R&D Systems, Oxford, UK) in 24-well plates for 6 days.

### Culture of immature DC with ECM proteins

To prepare ECM-coated plates, 10 µg/ml of fibronectin (from human plasma) or laminin (derived from human placenta, reported as isoform 511 in Gorfu et al [22]) (Sigma, Irvine, UK) were added into each well and the plates were incubated for 1.5 h at 37°C. Following incubation, the protein solution was removed by aspiration and the wells were washed twice with cold PBS. Immature DC were washed once, re-suspended in serum-free AIM V medium (Invitrogen, UK) and transferred to the coated wells. Control wells were normally coated with 1% BSA (Invitrogen) and contained immature DC cultured in the absence of laminin and fibronectin. These cultures were incubated for 48 h at 37°C. Cells were then used for the assays described below or analysed for expression of surface markers.

### Staining for cell surface markers

ECM-treated and control DC were harvested, washed twice with PBS, 0.5% BSA and sodium azide (PBA) and then stained for CD40 (clone LOB7/6, IgG2a, AbD Serotec), HLA-DR (clone Immu357, IgG1), CD80 (clone MAB104, IgG1), CD83 (clone HB15a, IgG2b), CD86 (clone HA5.2B7, IgG2b κ), Mannose Receptor [MR] (clone 3.29B1.10, IgG1, Beckman Coulter, High Wycombe, UK), DC-specific intercellular adhesion molecule-grabbing non-integrin [DC-SIGN] (clone DCN46, IgG2b κ, BD, Oxford, UK) and DEC205 (clone MG38, IgG2b, AbD Serotec) for 30 min at 4°C. Relevant isotype matched control antibodies were used, respectively. The cells were then washed with PBA and fixed in 0.5% formaldehyde in isotonic azide-free solution. Cells were usually analysed within 24 h.

### Flow cytometric analysis

Flow cytometry was performed using a Coulter EPICS XL-MCL (Beckman Coulter) and 50000 events were collected for each sample. Dead cells were excluded by forward and side scatter characteristics.

### Scanning electron microscopy (SEM) imaging

For SEM preparations, glass coverslips were coated with ECM proteins as described above. Immature DC were then added to the coverslips and incubated for 48 h at 37°C in a 12-well culture plate. Coverslips coated with 1% BSA were used as controls. Following incubation, the coverslips were removed from culture, washed once with PBS and stained for SEM as described elsewhere [23]. Images were obtained using a Jeol (Tokyo, Japan) JSM-6060LV SEM machine.

### Cell viability assay

Cell viability was measured using the CellTiter AQ One Solution Cell Proliferation™ (MTS) assay kit (Promega, Southampton, UK) according to the Manufacturer's instructions. Briefly, assays were performed in with reduced lighting, simply by the addition of 20 µl of CellTiter AQ reagent into the relevant samples in 100 µl of culture medium. These samples were then incubated in a humidified-atmosphere incubator at 37°C and with 5% CO_2_ for 90 min before the absorbance was read at 492 nm using a Optima FLUOstar® plate reader.

### Cell perimeter measurement

DC were cultured in FN or LMN-coated 24-well plates for 48 h. After incubation, light microscopy images were obtained using a 20X objective with a Leica microscope (Newcastle, UK). Images were then analysed using Leica QWin software measuring at least 50 cells in each condition.

### Re-stimulation of ECM-treated DC

After 48 h incubation in the presence or absence of ECM proteins, DC were harvested, washed once with AIM-V and re-plated in 96-well plates (2.5×10^5^ cells/well). LPS (200 ng/ml)(Sigma) or soluble CD40L (1 µg/ml) (Axxora, Bingham, UK) were added and the cells were further incubated for 48 h. DC were then harvested and their phenotype was analysed as described above.

### Endocytosis and blocking experiments

DC were washed once with PBS, re-suspended in RPMI and transferred into 1.6 ml eppendorfs (5×10^5^ cells/ml in 500 µl). Dextran-FITC (Sigma) was added to a final concentration of 1 mg/ml and the tubes were incubated for 1.5 h at 37°C or 4°C. Cells were harvested, washed twice with ice-cold PBS, re-suspended in isotonic azide-free solution and immediately analysed by flow cytometry. For blocking experiments, ECM-treated DC were pre-incubated with 1 mg/ml mannan (Sigma) for 20 min at 37°C before addition of dextran-FITC and subsequent analysis by flow cytometry. For assessing the uptake of MR and DC-SIGN specific ligands, ECM-treated DC were incubated for 1 hr at 37°C with either 20 µg/ml SO_4_-3-galactose-FITC or Lewis-X-FITC (Lectinity Corp, Moscow) respectively. Cells were then analysed by flow cytometry.

### Fluorescein-labeled CMV uptake

For CMV uptake, CMV pp65 protein (Miltenyi Biotec) was labeled with the Fluoro-Trap™ Fluorescein labeling kit (Innova Biosciences) according to the manufacturer's instructions. DC were incubated for 30 min at 37°C or 4°C with 10 µg/ml of fluorescein-labeled CMV protein before analysis by flow cytometry.

### Real Time PCR

DC were washed in ice-cold PBS and mRNA extraction and cDNA synthesis were performed using the µMACS™ one-step cDNA kit (Miltenyi Biotec) according to manufacturer's instructions. Real-Time PCR was performed in a Stratagene MxPro 3005P qPCR System with the Brilliant SYBR Green QPCR master Mix (Stratagene, La Jolla, USA). Primer sequences were as follow: GAPDH (forward) 5′-GAGTCAACGGATTTGGTCGT-3′, (reverse) 5′-GACAAGCTTCCCGTTCTCAG-3′; Mannose Receptor (forward) 5′-CGTTTACCAAATGGCTTCGT-3′, (reverse) 5′-CCTTGGCTTCGTGATTTCAT-3′; DC-SIGN (forward) 5′-CCAAAGGAGGAGACAAGCAG-3′, (reverse) 5′-GGACGACAGCTTCAGTGTGA-3′. Cycling was initiated at 95°C for 10 min, followed by 40 cycles of 95°C for 30 s, 55°C for 30 s and 72°C for 1 min. Samples were run in triplicate and relative expressions of MR and DC-SIGN were calculated using the comparative threshold cycle method normalized to GAPDH (2^−ΔΔCT^ mathematical model [24]).

### Antigen dependent T-cell proliferation

ECM-treated and ECM-untreated DC were loaded with CMV protein for 3 h before co-culturing with autologous memory T-cells, separated by negative selection from the whole T-cell fraction of PBMCs using a Pan T-cell Kit and CD45RO beads (Miltenyi Biotec). These co-cultures were incubated for different time points and T-cell proliferation was detected by H^3^-thymidine incorporation as described before [25]. Briefly, H^3^-thymidine (specific activity 24.0 Ci/mmol; Amersham Life Science, Buckingham, UK) was added to each well at a final concentration of 4 µCi/ml and cultures were continued for another 18 h. Cells were harvested and thymidine incorporation was measured in a scintillation counter. The proliferation index (PI) was calculated according to the following equation: PI  =  mean cpm of proliferating T cells cultured in the presence of ECM treated or un-treated DC–mean cpm of T cells cultured in the absence of DC.

### IFN-gamma quantification

IFN-gamma was quantified using an ELISA kit (R&D Systems, Oxford, UK) according to the manufacturer's instructions.

### DC-T cell conjugate analysis

DC-T cell conjugates were analysed by flow cytometry as described before [26], [27]. Briefly, DC (with and without treatment with FN or LMN) and T cells were labelled with PKH26 (Sigma) and Carboxyfluorescein Succinimidyl Ester (CFSE) (Invitrogen), respectively. DC and autologous T cells (in 1:2 DC:T cell ratio) were mixed, centrifuged for 5 min at 1000 rpm, to favour conjugate formation, and incubated for 45 min at 37°C. Pellets were then gently resuspended in 400 µl of PBS and immediately analysed by flow cytometry. The DC population was gated according to FSC/SSC parameters and conjugates were defined as CFSE+ cells in the DC population. To rule out phagocytosis or dye diffusion as a cause of double positive cells, conjugate formation was investigated on cells treated with 5 mM EDTA and vortexed for 2 min after incubation.

### Statistical analysis

The samples were defined by their mean values and standard deviation. Differences between the means were compared using the Student *t-*test and *p*<0.05 was considered significant.

## Results

### FN and LMN affect DC morphology

**Figure 1:**
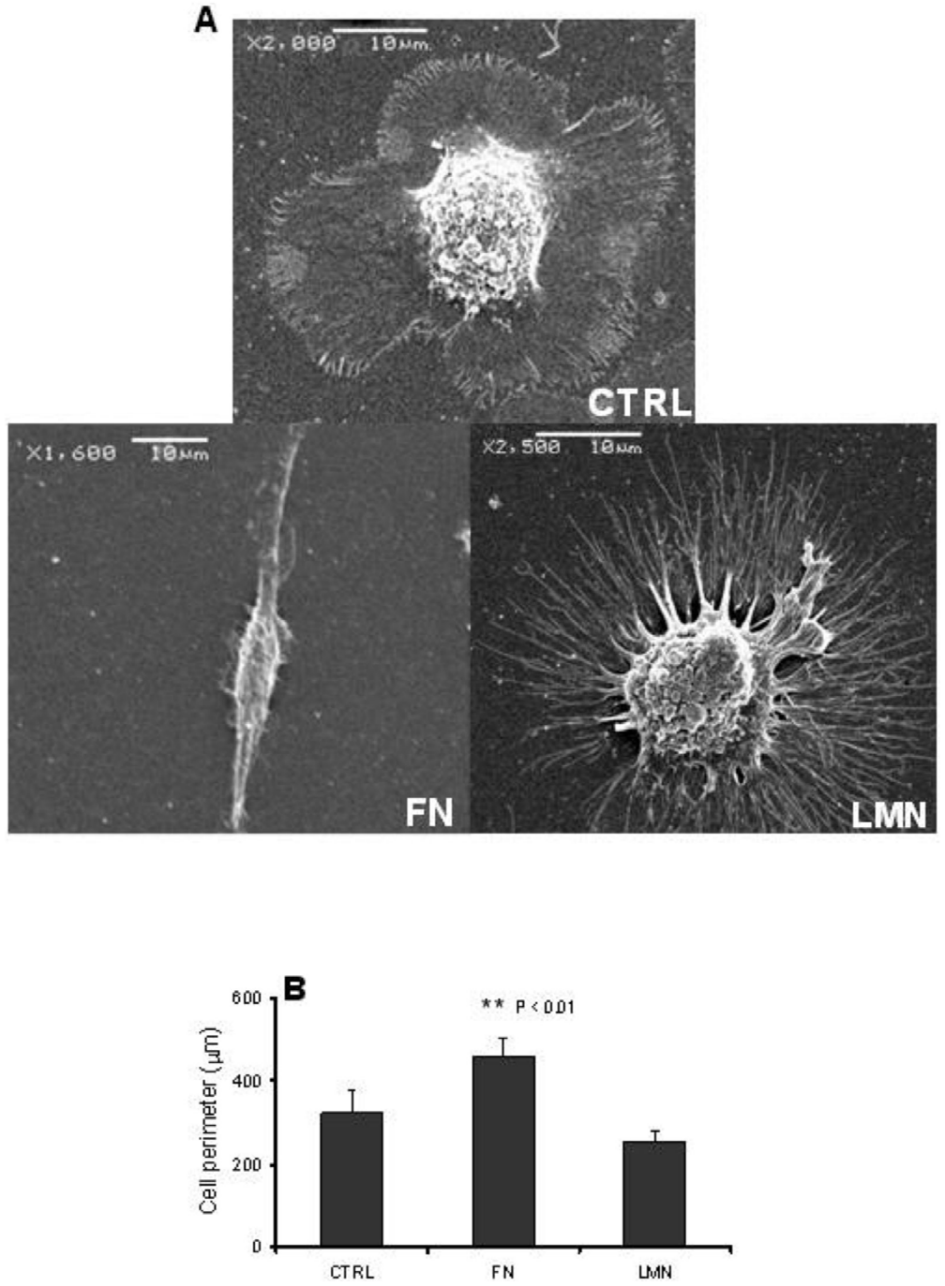
FN and LMN induce changes in DC morphology. *A*, SEM images of ECM-treated DC taken after 48 h of culture over FN or LMN-coated coverslips. Different magnifications were used to better visualize the whole cell morphology. *B*, Cell perimeter measurements based on light microscopy images. Results represent average values of at least 50 cells in each condition plus standard deviations.

To investigate the possible effect of ECM components on DC morphology, DC were cultured on FN and LMN-coated surfaces. The data show a notable difference in DC morphology after culturing cells on ECM coated surfaces (Fig. 1A). For instance, FN-treated DC developed long and broad dendrites, whereas LMN-treated DC developed long but thin dendrites. This difference was further confirmed by measuring total cell perimeter of DC, and this demonstrated a significant increase (P<0.01) in the surface area occupied by FN-treated cells compared to LMN-treated or untreated DC (Fig. 1B).

### FN and LMN affect dendritic cell phenotype

**Figure 2:**
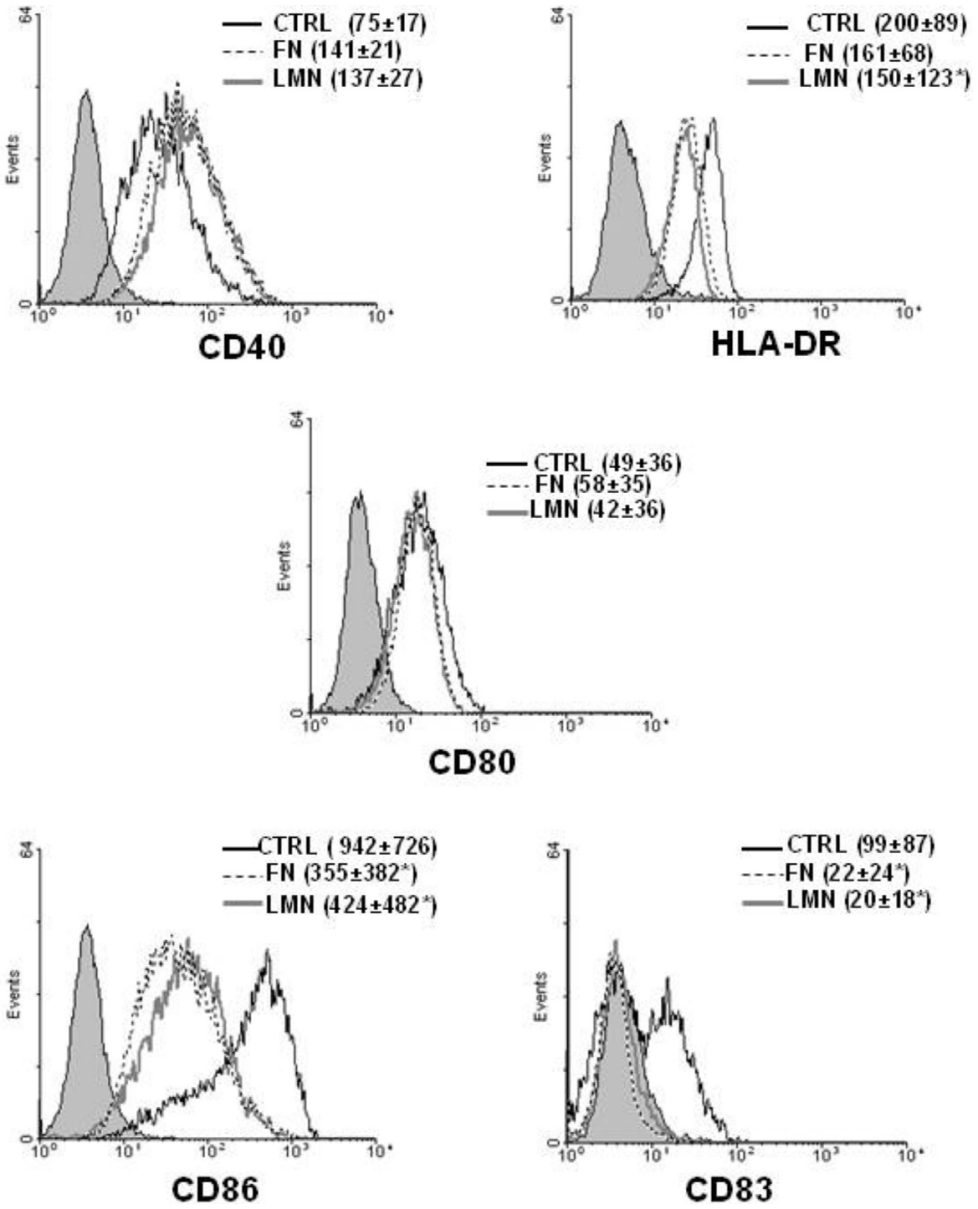
ECM-treatment causes a decreased expression of some DC maturation markers. DC membrane phenotype was analyzed after 48 h of culture in the presence of FN or LMN (both at 10 µg/ml) compared with immature DC. Filled histograms represent isotype controls, control (1% BSA treated) DC are depicted with the solid black line, FN treated DC are depicted with the dashed black line and LMN treated DC are depicted with the solid gray line. Results depict one representative out of four independent experiments. * P<0.05.

**Figure 3:**
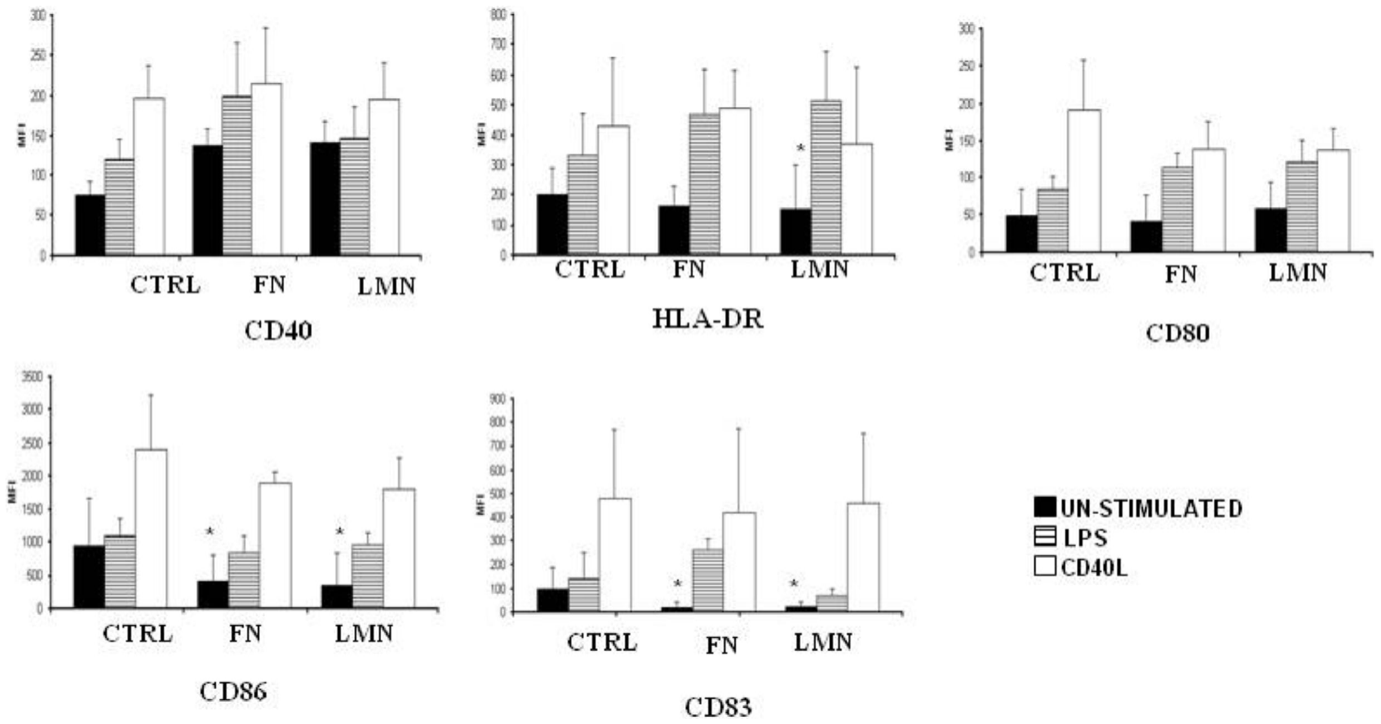
ECM-treated DC are able to fully mature after stimulation with maturation stimuli. Membrane phenotype of control, FN and LMN treated DC was analyzed after 48 h of incubation with 200 ng/ml LPS (n = 3) and 1 µg/ml CD40L (n = 4). Error bars represent standard deviations. MFI values for isotype controls for each antibody was deducted from the values shown. * P<0.05.

Immature DC were cultured in the presence of FN or LMN for 48 h and the DC phenotype was determined using flow cytometry. Fig. 2 shows that ECM components induced a decrease in the expression of some DC maturation markers (HLA-DR, CD83 and CD86), giving these cells a ‘more immature’ profile. Furthermore, using an MTS assay we compared the viability of DC cultured on BSA or ECM coated plates. These experiments showed no difference in cell viability between different culture conditions (data not shown). Considering the possibility that the effect of such ECM treatment could render DC un-responsive to further stimulation, we studied the maturation state of these cells after stimulation with LPS and CD40L. Fig. 3 shows that ECM-treated DC can recover from their ‘more-immature’ state and acquire a fully mature phenotype as evidenced by their increased expression of CD40, CD83 and HLA-DR.

### FN and LMN-treated DC show increased endocytic ability

**Figure 4:**
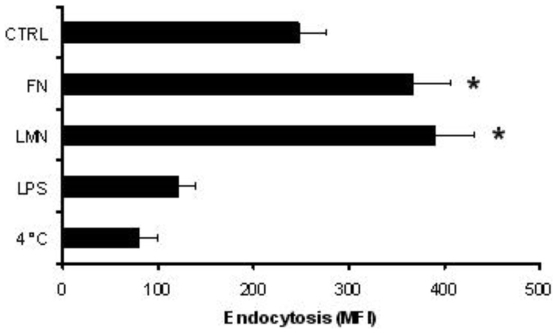
FN and LMN induce an increase in the endocytic ability of DC. Dextran-FITC uptake at 37°C was evaluated by flow cytometry. DC were incubated for 48 h in ECM-coated plates before performing the assays. n = 4 independent experiments. Error bars represent standard deviations. * P<0.001.

One of the characteristic features of immature DC is their high endocytic ability. After maturation, this capacity decreases, allowing DC to present the antigens they have captured in the periphery to T cells. We analyzed dextran-FITC uptake by DC after treatment with ECM components. Treatment with FN or LMN caused a significant increase (P<0.001) in the endocytic ability of DC (Fig. 4). These results are clearly in agreement with the phenotype data obtained earlier, which suggested a ‘more immature’ profile exhibited by ECM-treated DC. Interestingly, we did not observe any synergy between FN and LMN when a mixture of both proteins was used in these experiments (data not shown).

**Figure 5:**
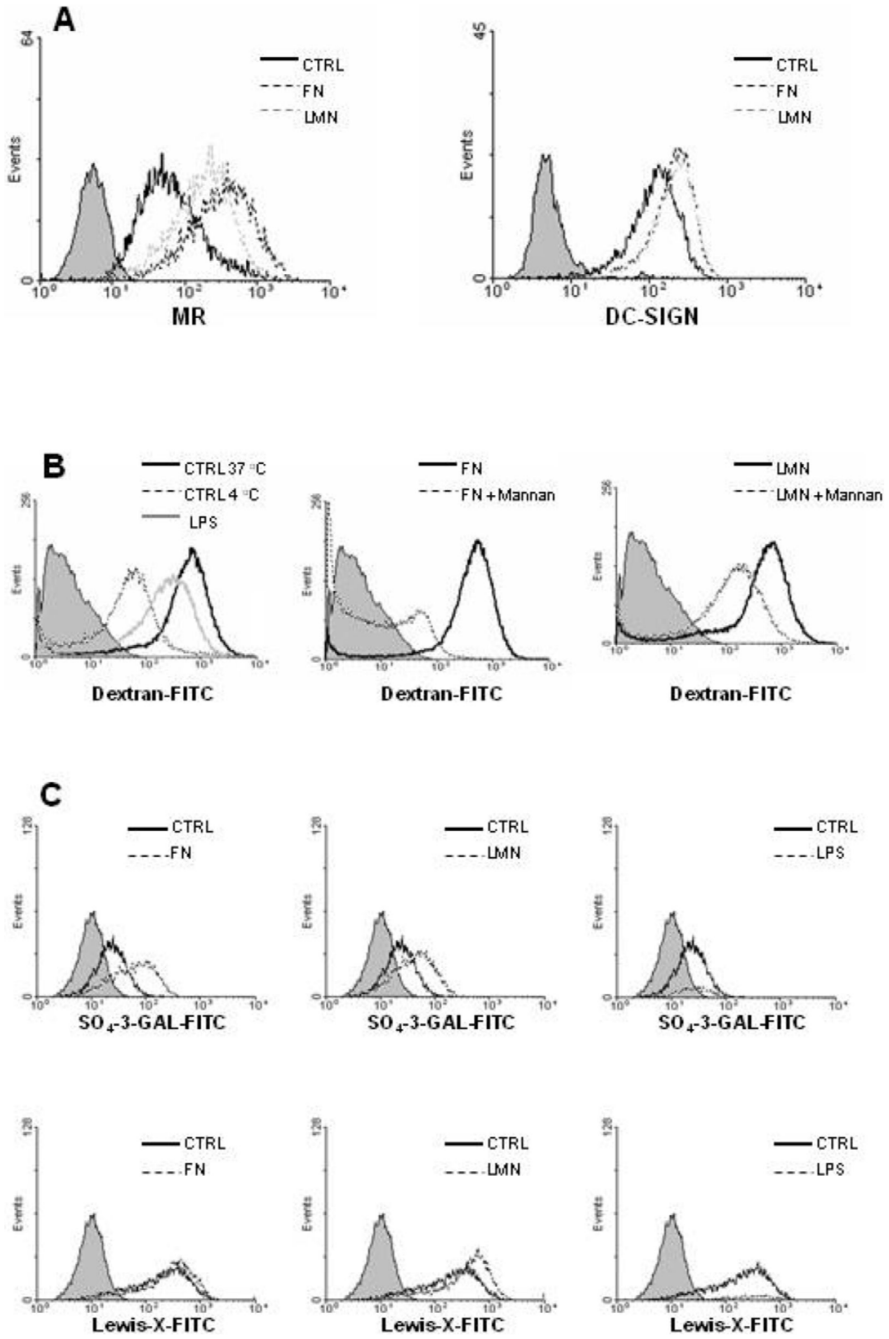
MR and DC-SIGN expression by DC is increased after ECM treatment. *A*, Following 48 h of culture in FN or LMN-coated plates (both 10 µg/ml), DC were stained for MR and DC-SIGN expression and analyzed by flow cytometry. Filled histograms (gray) represent isotype controls. *B*, The endocytic receptors MR and DC-SIGN were blocked using mannan before addition of dextran-FITC and analysis by flow cytometry. Filled histograms (gray) represent cells without dextran-FITC and results depict one representative out of three. *C*, The uptake of specific MR and DC-SIGN ligands by ECM-treated DC was also evaluated by flow cytometry. The sulphated sugar SO_4_-3-galactose-FITC is specifically bound by MR and Lewis-X-FITC by DC-SIGN. Filled histograms (gray) represent cells incubated without ligand. Results show one representative out of three independent experiments.

In order to ascertain that the increase in the endocytic capacity of ECM-treated DC was due to a change in the expression pattern of relevant endocytic receptors, we analysed membrane expression of MR, DC-SIGN and DEC205. We found that FN and LMN treatment induced up-regulation of MR and DC-SIGN (Fig. 5A), but not DEC205 (data not shown), expression on the surface of DC. Whilst this observation suggests that the increased endocytic capacity of LMN and FN treated DC is due to a higher expression of MR and DC-SIGN, dextran uptake could also be mediated through macropinocytosis. We therefore sought to further investigate this observation by performing blocking and uptake experiments using mannan, a common ligand for MR and DC-SIGN [28], as well as SO_4_-3-galactose (a sulphated sugar) [29] and Lewis-X [30], two specific ligands for MR and DC-SIGN, respectively. These experiments showed that the enhanced Dextran-FITC uptake demonstrated by FN and LMN treated DC was blocked in the presence of mannan (Fig. 5B), and FN and LMN treatment of DC led to an increase in the uptake of SO_4_-3-galactose and Lewis-X, which are MR and DC-SIGN specific ligands, respectively (Fig. 5C).

**Figure 6:**
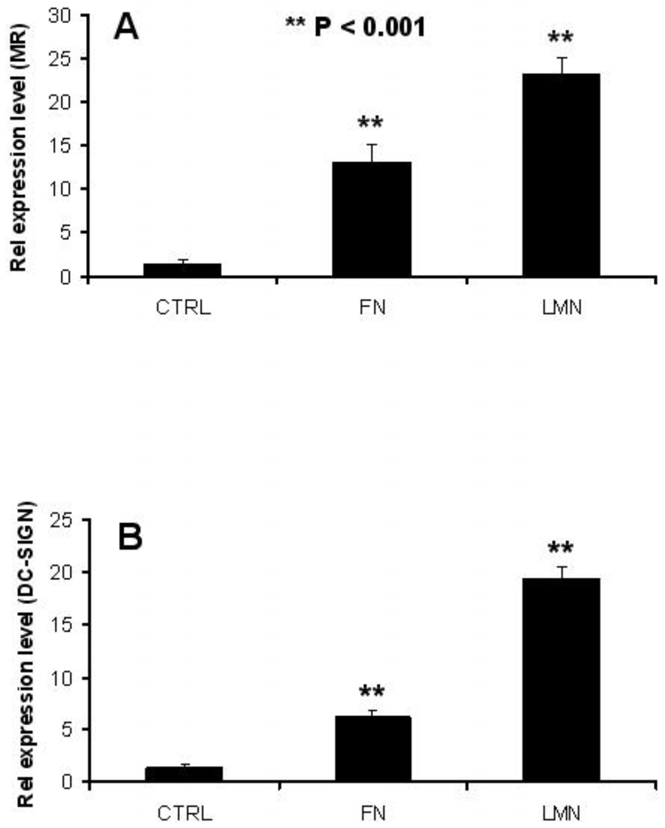
ECM induces MR and DC-SIGN up-regulation. After mRNA extraction and conversion to cDNA, MR and DC-SIGN expression was analyzed using Q-PCR. Relative expression levels of both genes were compared to that of a house-keeping gene (GAPDH). Results depict one representative out of three independent experiments.

This ECM-induced expression of MR and DC-SIGN could have been due to impaired shedding of these receptors or higher expression at gene level. Therefore, to establish if this was also accompanied by a change at transcriptional level, we performed RT-PCR experiments. These assays showed an increase in the relative expression of MR and DC-SIGN mRNA in FN and LMN-treated DC (Fig. 6).

### ECM-treated DC exhibit stronger T-cell interaction abilities and induce higher IFN-gamma production and T cell proliferation in an antigen-dependent manner

**Figure 7:**
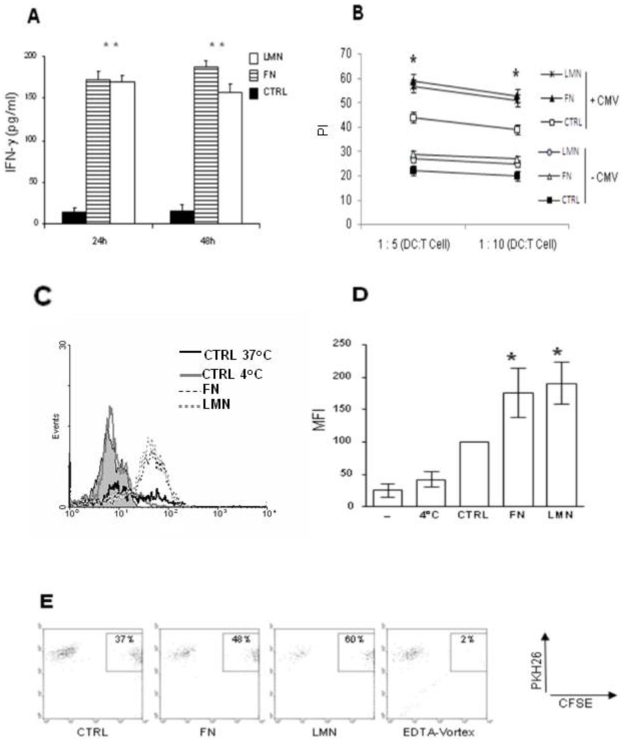
FN and LMN treatment influences the outcome of DC-T cell interactions. *A*, IFN-gamma production by memory T-cells after 24 and 48 h of co-culture with ECM-treated DC pre-loaded with CMV protein. *B*, T-cell proliferation assays using memory T-cells as responder cells. The proliferation index (PI) was calculated as described in the materials and methods. *C*, A representative histogram showing the uptake of fluorescein-labeled CMV protein by DC before and after treatment with ECM. Cells without CMV (filled histogram), cells at 4°C with CMV (solid gray line), control DC with CMV (solid black line), FN treated DC with CMV (dashed black line) and LMN treated DC with CMV (dashed gray line). *D*, CMV uptake by DC under different culture conditions. Data from figure 7C is represented as a histogram with n = 3. *E*, Analysis of conjugate formation between DC and T cells. PKH26-labeled DC were incubated with CFSE-labeled T cells for 45 min (ratio 1DC/2T cell). Conjugate formation was analyzed by flow cytometry by determining the CFSE+ cells amongst the DC population. To rule out phagocytosis or dye diffusion as a cause of double positive cells, conjugate formation was investigated on cells treated with 5 mM EDTA and vortexed for 2 min after incubation. Results depict data from one of three independent experiments. *P<0.05, ** P<0.001.

To study the possibility that ECM-treated DC behave differently in an antigen-specific system, we co-cultured FN or LMN-treated DC (loaded with a CMV envelope protein) with autologous memory T-cells and measured IFN-gamma production and T-cell proliferation. T-cells that were co-cultured with CMV-loaded ECM-treated DC produced significantly higher (P<0.001) amounts of IFN-gamma compared to CMV-loaded control DC (Fig. 7A). We also found that these T-cells have a higher proliferation index (P<0.05) compared to control cells (Fig. 7B). We then sought to determine the underlying mechanisms for this higher T-cell proliferation and IFN-gamma production. The analysis of DC phenotype revealed no significant changes in the expression of the co-stimulatory molecules CD80, CD86 and CD40 after CMV treatment (not shown). However, we found an up-regulation of CMV uptake in the presence of FN and LMN (Fig. 7C and 7D). Furthermore, the ability of DC to interact with T cells, which was assessed by DC/T conjugate formation, was found to be higher with the ECM treated DC compared to control cells (Fig. 7E).

## Discussion

ECM is the main component of the core structure of the LN, and ECM proteins are highly abundant in the LN paracortex, where DC encounter and activate T cells [20], [31], [32]. Furthermore, ECM components make up a high percentage of the protein content in the interstitial spaces of peripheral tissues, such as skin and respiratory epithelium, where immature DC reside. Yet, the interaction of DC with ECM components has been overlooked in many *in vitro* studies investigating human DC biology.

In this study, we have investigated the impact of two ECM components, FN and LMN, on the morphology, phenotype and functional properties of human monocyte-derived DC. The laminin family of glycoproteins consists of many isoforms. In this study, we have used a commercial preparation of laminin from the human placenta. A recent study by Gorfu et al, has reported this isoform to be laminin-511 (α5β1γ1) [22]. They also found laminin-511 to be the predominant isoform for the adhesion and migration of human blood lymphocytes followed by laminin 332 and 411 in the human lymph node. Interestingly, it has been shown that the basement membrane of high endothelial venules and reticular fibers of human lymph nodes express laminin isoforms 211, 311, 411 and 511 [22].

Our data clearly show that DC that have been in contact with either FN or LMN developed a different morphology compared to control DC. This was particularly noticeable in the number and length of their dendrites. FN-treated cells showed much longer dendrites and occupied a larger surface area, a finding which is in line with previous work showing that engaging β1-integrin (CD29) on the DC surface by FN leads to the formation of long dendrites in a protein specific manner and that no such effect is seen with collagens type I or II [33]. LMN-treated DC, however, developed much thinner but still long dendrites. It has been reported that LMN also promotes dendrite formation in other cell types, like melanocytes and neurons [34], but its effect on DC morphology had not been reported before. Dendrite formation is an important stage in DC development, since it allows DC to sample the microenvironment for microbes [35] and other potential danger signals. Thus, the longer DC dendrites, covering a larger surface area, seen here in the presence of FN or LMN could potentially lead to more efficient sampling of the microenvironment.

Immature DC are characterized by their high endocytic ability and low expression of certain membrane markers like CD40, HLA-DR and CD83. Upon antigenic stimulation, immature DC undergo a maturation process which leads to a number of phenotypical and functional changes. These include a marked decrease in their endocytic ability accompanied by up-regulation of co-stimulatory molecules like CD40, CD80, CD83 and CD86 [5]. Our data show that the presence of FN or LMN confers a ‘more immature’ phenotype on DC, as evidenced by their lower expression of maturation markers (CD83, HLA-DR) and co-stimulatory molecules (CD86). This is in agreement with earlier findings reporting that FN produces a slight decrease in the expression of some DC surface markers such as CD80, CD83 and CD86 [36]. More importantly, our data show a significant increase in the endocytic ability of FN or LMN treated cells, a characteristic feature of immature DC. Given that endocytosis of dextran and other similar structures is primarily mediated through the mannose receptor [MR] [37], we studied membrane expression of MR and other endocytic receptors such as DC-SIGN and DEC205, following treatment of DC with FN or LMN. This showed a significant up-regulation of MR and DC-SIGN after ECM treatment, with no such effect on DEC-205 expression.

These observations indicated that the enhanced endocytic ability of FN and LMN treated DC could be due to increased surface expression of MR and DC-SIGN. Indeed, we were able to confirm this association by using mannan, a common blocking reagent for MR and DC-SIGN. As expected, mannan led to suppression of Dextran uptake by FN and LMN treated DC. Furthermore, we used two specific ligands for MR and DC-SIGN, namely SO_4_-3-galactose [28], [29] and Lewis-X [28], [30], respectively. In those experiments, ECM-treated cells showed enhanced uptake of SO_4_-3-galactose and Lewis-X, further confirming the association between ECM-induced up-regulation of MR and DC-SIGN and the resulting enhanced endocytic ability of DC. Also, RT-PCR experiments showed that up-regulation of MR and DC-SIGN was associated with an increase in their mRNA levels, thereby indicating the contribution of *de novo* synthesis to their higher expression subsequent to FN and LMN treatment. However, the exact mechanism behind the higher MR and DC-SIGN expression is yet to be established, and it is possible that impaired shedding of DC-SIGN and MR at the cell surface could also contribute to this phenomenon.

Recognition and binding to ECM elements is mainly mediated by integrin receptors, many of which are expressed by DC [38]. It has been shown that signalling through these receptors after cell-cell contact or cell-ECM interaction leads to activation of different signalling pathways [39], [40]. Thus, the demonstration here that the control of genes encoding the synthesis of MR and DC-SIGN, as two key endocytic receptors [41], is affected by ECM components is a novel observation and one that highlights the importance of incorporating ECM in studies of DC cultures and DC-T cell co-cultures.

Although ECM-treated DC did not show any significant difference in their ability to induce T cell proliferation in mixed lymphocyte reaction experiments compared to control DC, interestingly T-cells that were co-cultured with CMV envelope protein-loaded and ECM-treated DC produced higher amounts of IFN-gamma and showed a higher proliferation rate. Whilst we did not find any significant differences in the expression of the co-stimulatory molecules CD80, CD86, and CD40, we observed an increase in CMV uptake by DC treated with FN or LMN. This extra loading, combined with stronger ability of ECM-treated DC to form conjugate with T cells, could account for the observed higher proliferation and IFN-gamma secretion by T-cells.

Whilst our data indicate that laminin and fibronectin could affect different aspects of DC phenotype and function, the ECM impact on DC endocytic capacity and their ability to cross-talk with T cells seem to be the dominant effects. This is clearly shown in our endocytosis assays and DC-T cell co-culture experiments where ECM treated DCs establish more efficient link with T cells and support the production of significantly higher levels of IFN-gamma.

*In vivo*, DC migration, maturation and interaction with T cells happen in the presence of different ECM components. FN and LMN are two abundant ECM proteins present in tissue basement membranes and interstitial spaces, as well as in the T cell area of LN paracortex [20], [31], [32]. The majority of studies on DC biology are carried out in the absence of ECM, and this is not ideal as this study will have shown. The data described here clearly show the significant impact of ECM on key DC functions. It is, therefore, reasonable to suggest that the inclusion of ECM components should be considered in future *in vitro* studies investigating DC biology. Of course, this would only be a first step towards better simulation of *in vivo* events, and other equally important factors, such as the 3D structure of different tissues and the interdependency between effector and structural cells, should also be considered.
